# Learning From Resident Councils: How Residents Council Can Provide Vital Data for Internal and External Research

**DOI:** 10.1093/geroni/igaf122.1983

**Published:** 2025-12-31

**Authors:** Mairead Painter, Daniel Beem, Alice Bonner

**Affiliations:** State of Connecticut, Hartford, Connecticut, United States; State of Connecticut, Hartford, Connecticut, United States; Institute for Healthcare Improvement, Boston, Massachusetts, United States

## Abstract

Resident Councils serve as vital sources of process and outcomes data in nursing homes, offering researchers and organizational leaders valuable insights into care quality, resident well-being, and systems-level improvement opportunities. This paper explores structured approaches that enhance meaningful resident participation, generating actionable data for quality improvement. We will first examine Resident Councils as data sources, discussing methods for inclusively collecting and analyzing resident-driven feedback—including process measures such as engagement levels, issue tracking, and advocacy effectiveness. Next, we will explore how nursing homes can integrate Resident Council insights and direct participation into their federally required Quality Assurance and Performance Improvement (QAPI) programs to drive measurable improvements in care delivery, resident satisfaction, and operational efficiency. Finally, we will consider broader research and policy implications, identifying how resident council engagement can inform quality benchmarks, innovative long-term care research efforts and approaches, and policy initiatives at local, state, and national levels. The paper will highlight lessons learned from ongoing initiatives—specifically the Moving Forward Nursing Home Quality Coalition and its ongoing Strengthening Resident Councils pilot—and showcase real-world examples of how resident voices contribute to evidence-based quality improvement. By leveraging Resident Councils as an inclusive, action-oriented, and valuable source of key information, nursing homes can foster a more responsive, resident-directed approach to care.

